# Prevalence and risk factors of hypertension and diabetes among persons living with HIV in Zambia: results of a national facility‐based cross‐sectional survey

**DOI:** 10.1002/jia2.70051

**Published:** 2025-10-07

**Authors:** Chomba Mandyata, Sivile Suilanji, Samuel Bosomprah, Paul Somwe, Cosmas Zyambo, Mwiche Musukuma, Aggrey Mweemba, Malizgani Paul Chavula, Chipefwe Sichilima, Phoebe Bwembya, Mpanji Siwingwa, Richard Chibale, Henry Phiri, Joseph Zulu, Halwindi Hikabasa, Wilbroad Mutale

**Affiliations:** ^1^ School of Public Health University of Zambia Lusaka Zambia; ^2^ Center for Infectious Disease Research in Zambia (CIDRZ) Lusaka Zambia; ^3^ Ministry of Health Lusaka Zambia; ^4^ Department of Biostatistics School of Public Health University of Ghana Accra Ghana

**Keywords:** non‐communicable diseases, HIV, blood pressure, hypertension, glycosylated A1c, prediabetes, diabetes mellitus

## Abstract

**Introduction:**

Despite growing evidence on the rising burden of non‐communicable diseases (NCDs) in sub‐Saharan Africa, the national prevalence of hypertension, prediabetes and diabetes among persons living with HIV (PLHIV) in Zambia is largely unknown. This study aimed to determine the national prevalence of hypertension and diabetes mellitus and their associated risk factors among adult PLHIV in Zambia.

**Methods:**

We conducted a cross‐sectional study in 149 antiretroviral therapy (ART) clinics located in 52 rural and urban districts in Zambia based on the adapted World Health Organization (WHO) STEPwise approach to NCD risk factor Surveillance (STEPS) and the Zambia Population‐Based HIV Impact Assessment (ZAMPHIA) questionnaire. We used proportional to size sampling to select districts and clinics, targeting 5775 PLHIV. Data was collected from 1 October 2023 to 30 November 2023. We estimated the prevalence of hypertension and diabetes mellitus and used robust Poisson regression to analyse associations with socio‐demographic, behavioural and HIV‐related risk factors, and reported prevalence ratios (PR).

**Results:**

In the final analysis, we included a total of 5204 participants from 52 districts and 149 ART clinics countrywide: 67.2% were female, and 71.3% were from urban areas. The prevalence of hypertension, prediabetes and diabetes was 22.5% (95% confidence interval [CI]: 21.3−23.6), 26.7% (CI: 25.5−27.9) and 12.5% (CI: 11.6−13.4), respectively. In the multivariable model, being 30–44 (PR = 2.1; CI: 1.5−2.9), 45–49 (PR = 3.3; CI: 2.4−4.7) and 60 years or older (PR = 4.7; CI: 3.3−6.8) compared to those aged 18–29; widowed, divorced or separated individuals compared to those never married; being overweight (PR = 1.4; CI: 1.2−1.5) and obese (PR = 1.9; CI: 1.6−2.1) compared to normal weight PLHIV was associated with hypertension. College or university‐educated PLHIV (PR = 2.1; CI: 1.3−3.4), compared to those with no formal education; and those with high total cholesterol ≥6.2 mmol/l (PR = 2.2; CI: 1.4−3.6), versus desirable total cholesterol (<5.2 mmol/l); being overweight (PR = 1.4; CI: 1.1−1.6) and obese (PR = 1.6; CI: 1.3−2.0), compared to those with normal weight, showed a significant association with diabetes mellitus.

**Conclusions:**

The prevalence of hypertension and diabetes mellitus among PLHIV in Zambia was notably high. This underscores the need for immediate and robust intervention strategies to mitigate the high prevalence of hypertension and diabetes mellitus, along with their associated risk factors, particularly within this vulnerable demographic.

## 1 INTRODUCTION

Several decades on since its first discovery, the human immunodeficiency virus (HIV) acquisition in humans remains a significant public health problem of international concern. Sub‐Saharan Africa (SSA) remains the most affected region [1]. The introduction and sustained scaling up of the highly active antiretroviral therapy in the early 2000s, the test and treat approach to HIV treatment, coupled with other health system strengthening interventions, have significantly contributed to Zambia achieving two [2] of the three UNAIDS 95‐95‐95 goals for the elimination of AIDS by 2030. Persons living with HIV (PLHIV) adherent to antiretroviral therapy (ART) are now experiencing an extended life expectancy [3, 4]. While this is good in and of itself, PLHIV on ART are now predisposed to ageing, behavioural and lifestyle‐associated diseases, especially non‐communicable diseases (NCDs), including cardiovascular diseases (CVDs) [5, 6, 7, 8, 9], chronic kidney failure [10, 11, 12], depression [13, 14, 15], diabetes mellitus [16, 17], hyperlipidaemia [18] and chronic respiratory diseases [19].

Hypertension and diabetes mellitus are independently associated with CVDs such as stroke, ischemic heart failure, coronary artery disease and peripheral artery disease [20, 21, 22, 23, 24]. Living with HIV itself, long‐term use of ART, ageing and lifestyle‐related risk factors have been linked to the onset of NCDs [25]. Exposure to ART is associated with the redistribution of body fat as well as cardiometabolic conditions such as raised blood pressure, prediabetes, dyslipidaemia and insulin resistance [26]. Chronic inflammation and immune functional abnormalities brought about by the HIV acquisition have also been associated with the development of metabolic syndrome, such as dyslipidaemia, diabetes mellitus and atherosclerosis [27, 28, 29]. Metabolic syndrome has been reported to be more prevalent in PLHIV than in the HIV‐negative population [30]. Dolutegravir‐based regimens have been associated with overweight and obesity [31, 32], and hyperglycaemia [33, 34]—catalysts for hypertension and diabetes mellitus. Large swaths of the SSA population remain undiagnosed with hypertension, and among the diagnosed, only a quarter are on treatment, and of these, only about a tenth are controlled [35].

NCDs are socio‐economically costly to manage. If left to metastasize within societies and without effective interventions, they can significantly drain the already scarce resources required for uplifting lives, especially in low‐ and middle‐income countries (LMICs) [1, 36]. Understanding the national burden of hypertension and diabetes mellitus and their associated risk factors among PLHIV would inform appropriate interventions to address the growing complexity of managing co‐morbidity of HIV and NCDs in Zambia and other LMICs [37], as well as to maintain and advance the ongoing fight against HIV [38]. This study aimed to establish the nationwide prevalence of hypertension and diabetes mellitus and their associated risk factors among PLHIV in Zambia.

## 2 METHODS

### 2.1 Study design and setting

This study utilized a two‐stage stratified cross‐sectional survey design conducted at health facilities offering ART services. In the first stage, we stratified the facilities by province (10 provinces) and by rural‐urban classification, resulting in 20 strata. We used the probability proportional to size (PPS) approach in sampling health facilities. Health facilities with large HIV populations (>2500) in care had a higher likelihood of being sampled compared to smaller facilities. Health facilities were eligible for inclusion in the sampling pool if they offered ART services. The sample size calculation employed a precision sampling approach to estimate the prevalence of hypertension and diabetes mellitus among people living with HIV in Zambia, ensuring a defined level of precision. The prevalence of hypertension in the general population was estimated to be 19% [39]. We expected the prevalence to be higher in the adult PLHIV population. Assuming a prevalence of 50% (conservative estimate), a sample size of 385 was calculated to yield a two‐sided 95% confidence interval with a margin of error of 0.10, ensuring sufficient precision in the estimates. To ensure adequate representation across age and sex groups, as well as to allow for urban‐rural disaggregation, we multiplied the sample size of 385 by the number of domains. The number of domains was determined by considering both males and females, as well as four age groups (18−29, 30–44, 45–59 and 60+ years), resulting in eight study population groups. This approach gave us a sample size of 3080. We further adjusted for a design effect of 1.5 to account for the cluster sampling, resulting in a sample size of 4620 (i.e. 3080 × 1.5). To account for a 20% non‐response rate, we adjusted our sample size to 5775 (4620/0.8) adult PLHIV on ART countrywide. The total sample of 5775 adult PLHIV was allocated proportionally across the 20 strata. Based on a fixed sample size of 35 adult PLHIV patients per clinic, 165 clinics (i.e. 5775/35) were selected from the pool of eligible clinics using PPS. The 165 clinics were allocated to the 20 strata proportionately to size, where size was defined as the total number of adult PLHIV on ART in the clinic. In the second stage, a fixed sample of 35 eligible HIV patients was systematically selected and surveyed per day at each clinic. Participants were randomly sampled throughout the morning and afternoon sessions to minimize selection bias.

### 2.2 Study participants

The study was conducted between 1 March 2023 and 30 November 2023. All PLHIV who were 18 years or older and were accessing ART services at the sampled health facility at the time of the survey were eligible for enrolment. To reduce confounding emanating from gestational diabetes and pregnancy‐induced hypertension in the study results, we opted to exclude pregnant women and breastfeeding mothers.

### 2.3 Data collection

ART nurses and laboratory technicians from each sampled facility were brought to Lusaka, the capital city of Zambia, for a 2‐day intensive training on study procedures conducted in March and September 2023. ART nurses were responsible for screening and enrolling participants, obtaining informed consent, administering the behavioural questionnaire and collecting physical measurement results. The laboratory technicians collected urine and blood samples, ensuring their proper handling and transportation to the central laboratory in accordance with study protocols. Informed consent was obtained by first informing the potential participant about the study's purpose, procedures, benefits and risks of taking part in the survey. Participants were then asked if they would be willing to participate in the study. If agreeable, they were asked to sign the informed consent form. Data collection was conducted between 1 October 2023 and 30 November 2023.

#### 2.3.1 Behavioural and lifestyle assessment

The study utilized an adapted questionnaire from the World Health Organization STEPwise approach to NCD risk factor surveillance (WHO STEPs) questionnaire [40] and the Zambia Population‐Based HIV Impact Assessment (ZAMPHIA) questionnaire [2].

#### 2.3.2 Anthropometric measurements

All physical measurements were recorded with precision to the nearest 0.1 cm for height, waist and hip circumference, and to the nearest 0.1 kg for weight. Measurements were captured as follows:

- Weight and height were measured using a pre‐calibrated weighing scale and stadiometer, respectively. Participants wore light clothing and were barefoot during the measurements.
- Blood pressure measurements were taken three times with a 5‐minute rest between each measurement using a digital blood pressure machine [41, 42].

### 2.4 Biochemical measurements

Consenting participants had 4 ml of whole blood collected in vacuum tubes containing ethylenediamine tetra acetic acid anticoagulant (purple top) and mixed thoroughly. Blood specimens were collected by a trained phlebotomist via venipuncture, following universal precautions to ensure safety and sterility [43]. All samples were analysed for glycosylated haemoglobin A1c (HbA1c) and total cholesterol using the AU480 Beckman Coulter chemistry analyser at the University Teaching Hospital Central laboratory in Lusaka, Zambia. Any results with abnormal values were reported to the study team for appropriate intervention.

### 2.5 Data entry and management

Each research associate entered all survey data except the biochemical data (entered by laboratory personnel) using a handheld device preloaded with the questionnaires via the Kobocollect mobile application. A designated data management specialist at the Ministry of Health Headquarters in Lusaka, Zambia, managed the study database. Barcodes were used to link the survey participants to the laboratory samples and results.

### 2.6 Statistical analysis

Participant characteristics were summarized by sex and area of residence using frequency and proportions for categorical variables. Measurement cutoffs applied are shown in Table 1. The prevalence of hypertension and diabetes, associated risk factors and corresponding 95% confidence intervals (CI) were estimated for each sex and area of residence. We used the Chi‐square or Fisher's exact test to compare proportions between groups. We applied robust Poisson regression [44] to determine the prevalence ratio (PR) of factors associated with hypertension or diabetes mellitus. The level of significance was set at 0.05. To identify factors independently associated with hypertension or diabetes mellitus, we considered factors with a *p*‐value <0.10 in univariable analyses for inclusion in the multivariable analysis. All variables with a *p*‐value >0.1 in the univariable analyses were not included in the multivariable analyses. All analyses were adjusted for survey design features, including clustering, sampling weight and stratification. All analyses used Stata 17 MP (StataCorp, College Station, TX, USA).

**Table 1:** Measurement cuff‐offs

Measure	Cutoff	
**Body mass index (BMI)**		
**Underweight**	≤18.5 kg/m^2^	
**Normal weight**	18.5–24.9 kg/m^2^	
**Overweight**	25–29.9 kg/m^2^	
**Obese**	≥30 kg/m^2^	
**Glycosylated haemoglobin A1c (HbA1c)**		
**Normal**	≤5.6%	
**Prediabetes**	5.7−6.4%	
**Diabetes mellitus**	≥ 6.5%	
**Blood pressure** [41]	**Systolic blood pressure (SBP)** ^a^	**Diastolic blood pressure (DBP)** ^a^
**Not hypertensive**	≤139 mmHg	≤89 mmHg
**Hypertensive**	≥140 mmHg	≥90 mmHg

^a^ Mean of the last two blood pressure measurements.

### 2.7 Ethical considerations

Ethical approval (REF. NO. 3007‐2022) was granted by the University of Zambia Biomedical Research Ethics Committee (UNZABREC).

## 3 RESULTS

### 3.1 Socio‐demographic characteristics

Complete socio‐demographic, behavioural factors and HIV characteristics of the surveyed PLHIV are presented in Table 2. A total of 5204 participants were included in the final analysis. In total, 3495 (67.2%) were females, and 3713 (71.4%) came from urban areas, respectively. The mean age was 42.9 years (standard deviation [SD] 11.8 years). A larger proportion of the surveyed PLHIV were in the age range (in years) of 30–44 (42.5%), followed by those aged 45–59 (35.7%). More than half (57.1%) of the surveyed PLHIV were married or in a cohabiting relationship. More females (37.2%) were either widowed, separated or divorced than males (14.9%). More males (49.2%) had a secondary level education than females (41.7%). PLHIV residing in urban areas (49.1%) had a secondary level education compared to those who reside in rural areas (33.9%).

**Table 2:** Background characteristics of participants

		Sex		Location	
	Total *N* = 5204	Females *N* (%) = 3495 (67.16)	Males *N* (%) = 1709 (32.84)	Rural *N* (%) = 1491 (28.65)	Urban *N* (%) = 3713 (71.35)
	% of column total (95% CI)	% of column total (95% CI)	% of column total (95% CI)	% of column total (95% CI)	% of column total (95% CI)
Age [SD]	42.9 [11.8] (42.6−43.3)	41.9 [11.5] (41.5−42.3)	45.0 [12.3] (44.4−45.6)	43.4 [11.4] (42.8−44.1)	42.7 [12.0] (42.3−43.1)
**Age group (years)**					
18–29	13.4 (12.5−14.4)	15.0 (13.8−16.2)	10.2 (8.9−11.7)	13.5 (11.8−15.3)	13.4 (12.4−14.6)
30–44	42.5 (41.17−43.9)	44.3 (42.6−46.0)	38.9 (36.5−41.2)	40.0 (37.5−42.5)	43.7 (42.1−45.4)
45–59	35.7 (34.4−37.0)	33.6 (32.1−35.4)	39.8 (37.5−42.2)	37.0 (34.6−39.5)	35.0 (33.5−36.6)
60+	8.4 (7.6−9.2)	7.02 (6.2−7.9)	11.1 (9.6−12.7)	9.6 (8.2−11.2)	7.8 (7.0−8.7)
**Marital status**					
Never married	13.0 (12.1−13.9)	12.5 (11.5−13.7)	13.9 (12.4−15.6)	10.4 (8.9−12.0)	14.2 (13.1−15.4)
Married/cohabiting	57.1 (55.8−58.5)	50.3 (48.6−52.0)	71.1 (68.9−73.3)	60.1 (57.6−62.6)	55.7 (54.1−57.3)
Widowed/separated/divorced	29.9 (28.6−31.2)	37.2 (35.6−38.8)	14.9 (13.3−16.8)	29.5 (27.2−31.8)	30.1 (28.6−31.6)
**Education level**					
None	5.0 (4.5−5.7)	5.9 (5.1−6.7)	3.4 (2.6−4.4)	8.5 (7.2−10.0)	3.4 (2.9−4.1)
Less than primary	17.9 (16.9−19.0)	20.1 (18.8−21.4)	13.5 (11.9−15.2)	25.6 (23.5−27.8)	14.2 (13.1−15.4)
Primary	24.5 (23.3−25.7)	25.8 (24.3−27.3)	21.9 (19.9−24.0)	28.5 (26.3−30.8)	22.6 (21.3−24.0)
Secondary	44.2 (42.8−45.5)	41.7 (40.1−43.3)	49.2 (46.8−51.6)	33.9 (31.6−36.3)	49.1 (47.5−50.7)
College+	8.4 (7.7−9.1)	6.6 (5.8−7.4)	12.0 (10.6−13.6)	3.5 (2.7−4.6)	10.7 (9.7−11.7)
**Physical measurements**					
Height [SD]	161.9 [9.2] (161.6−162.1)	158.9 [7.9] (158.6−159.1)	168.0 [8.6] (167.6−168.4)	161.0 [9.8] (160.5−161.4)	162.3 [8.8] (162.0−162.6)
Weight [SD]	62.4 [12.8] (62.0−62.7)	62.3 [13.6] (61.8−62.7)	62.6 [11.0] (62.1−63.1)	60.1 [11.1] (59.5−61.0]	63.5 [13.4] (63.0−63.9)
**Body mass index (kg/m^2^)**					
Mean BMI index	23.9 [5.0] (23.7−24.0)	24.7 [5.2] (24.5−24.9)	22.2 [3.9] (22.0−22.4)	23.3 [4.6] (23.0−23.5)	24.1 [5.1] (24.0−24.3)
Underweight	9.4 (8.6−10.2)	7.3 (6.5−8.3)	13.5 (11.9−15.3)	10.7 (9.3−12.4)	8.7 (7.8−9.7)
Normal weight	57.9 (56.5−59.2)	53.2 (51.5−54.9)	67.5 (65.2−69.7)	61.9 (59.4−64.3)	56.0 (54.3−57.6)
Overweight	22.2 (21.1−23.4)	25.8 (24.3−27.3)	14.9 (13.3−16.7)	19.3 (17.4−21.4)	23.6 (22.2−25.0)
Obese	10.5 (9.7−11.4)	13.7 (12.6−14.9)	4.1 (3.26−5.1)	8.0 (6.8−9.5)	11.7 (10.7−12.8)
**Tobacco use**					
Current smokers	9.9 (9.1−10.8)	3.8 (3.2−4.6)	22.2 (20.3−24.3)	10.4 (8.9−12.1)	9.4 (8.6−10.4)
Current consumer	61.6 (59.5−63.7)	53.0 (50.0−56.0)	70.8 (67.9−73.6)	58.5 (54.4−62.5)	62.4 (60.0−64.7)
Consumer 6 or more standard drinks per sitting	22.7 (20.5−25.1)	16.1 (13.3−19.4)	28 (24.7−31.5)	19.4 (15.4−24.3)	23.8 (21.2−26.5)
**Blood pressure measurements**					
Mean SBP [SD]	122.2 [18.1] (121.7−122.7)	121.7 [18.4] (121.1−122.4)	123.1 [17.4] (122.3−123.9)	121.1 [16.7] (120.2−121.9)	122.7 [18.7] (122.1−123.3)
Mean DBP [SD]	78.0 [0.2] (77.7−78.4)	78.0 [0.2] (77.6−78.4)	78.2 [0.3] (77.59−78.7)	76.9 [0.3] (76.3−77.5)	78.6 [0.2] (78.2−79.0)
SBP ≥ 140 mmHg and/or ≥DBP 90 mmHg/or currently on medication	22.5 (21.3−23.6)	21.9 (20.5−23.3)	23.7 (21.7−25.8)	19.0 (17.2−21.0)	24.1 (22.7−25.5)
**Systolic blood pressure category**					
Normal^a^	38.5 (37.2−39.8)	39.2 (37.6−40.9)	37.1 (34.7−39.4)	44.0 (41.6−46.5)	35.8 (34.3−37.4)
Elevated^b^	41.8 (40.5−43.2)	41.9 (40.3−43.6)	41.5 (39.2−44.0)	39.0 (36.6−41.5)	43.2 (41.5−44.8)
Stage 1^c^	13.3 (12.3−14.2)	12.4 (11.3−13.6)	15.0 (13.3−16.8)	10.8 (9.4−12.5)	14.4 (13.3−15.6)
Stage 2^d^	6.4 (5.8−7.1)	6.4 (5.7−7.3)	6.4 (5.3−7.7)	6.1 (5.0−7.4)	6.6 (5.8−7.5)
**History of raised blood pressure**					
No	48.4 (45.6−51.2)	44.8 (41.3−48.4)	55.1 (50.3−59.9)	50.2 (44.8−55.7)	47.7 (44.5−51.0)
Yes	51.6 (48.8−54.4)	55.2 (51.6−58.7)	44.9 (40.1−49.8)	49.8 (44.3−55.2)	52.3 (49.0−55.5)
**On anti‐hypertensive medication**					
No	66.1 (63.4−68.8)	61.7 (58.1−65.1)	74.6 (70.0−78.6)	68.1 (62.6−73.2)	65.4 (62.2−68.5)
Yes	33.9 (31.2−36.6)	38.3 (34.9−41.9)	25.4 (21.4−30.0)	31.9 (26.8−37.4)	34.6 (31.5−37.8)
**On medication and blood pressure control**					
No	69.4 (64.75−74.74)	69.1 (63.6−74.1)	70.3 (60.4−78.6)	67.5 (57.9−75.9)	70.1 (64.7−75.00)
Yes	30.6 (26.26−35.25)	30.9 (25.9−36.4)	29.7 (21.4−39.6)	32.5 (24.1−42.1)	29.9 (25.0−35.3)
**Time on ART**					
≤1 year	14.8 (13.8−15.8)	14.7 (13.5−15.9)	15.0 (13.4−16.8)	16.4 (14.7−18.3)	14.0 (12.9−15.2)
2−5 years	28.3 (27.1−29.6)	27.8 (26.4−29.4)	29.3 (27.1−31.5)	29.4 (27.2−31.7)	27.8 (26.3−3.2)
6−9 years	24.3 (23.1−25.5)	24.8 (23.3−26.2)	23.2 (21.3−25.4)	23.4 (21.3−25.6)	24.7 (23.3−26.1)
10+ years	32.6 (31.4−33.9)	32.7 (31.2−34.3)	32.4 (30.2−34.7)	30.8 (28.6−33.1)	33.5 (32.0−35.1)
**HIV drug regimen**					
Dolutegravir‐based regimen	96.4 (96.0−96.8)	96.6 (96.0−97.1)	96.0 (95.01−96.7)	93.7 (93.0−94.3)	97.7 (97.2−98.2)
Other	3.6 (3.2−4.0)	3.39 (2.9−3.9)	4.0 (3.2−5.0)	6.3 (5.7−7.0)	2.3 (1.8−2.8)
Mean days of HIV drug dispensation [SD]	152.5 [50.9] (151.1−153.8)	154.0 [48.1] (152.4−155.6)	152.3 [51.1] (149.8−154.7)	151.4 [49.0] (149.0−153.8)	154.4 [48.9] (152.9−156.0)
DSD (≥ 180 days of ARV drug supply)	74.1 (72.9−75.2)	74.1 (72.6−75.6)	74.0 (71.8−76.1)	73.6 (71.5−75.6)	74.3 (72.8−75.7)
**HbA1c**					
Mean HbA1c [SD]	5.6 [1.2] (5.6−5.6)	5.5 [1.2] (5.5−5.6)	5.6 [1.2] (5.6−5.7)	5.4 [1.0] (5.4−5.5)	5.7 [1.3] (5.6−5.7)
HbA1c category					
Normal	65.2 (63.9−66.5)	69.3 (67.0−71.5)	63.2 (61.6−64.8)	68.8 (66.6−70.6)	63.4 (61.8−65.0)
Prediabetes	26.7 (25.5−27.9)	27.0 (25.6−28.6)	25.8 (23.7−28.1)	25.5 (23.5−27.6)	27.2 (25.8−28.7)
Diabetes	12.5 (11.6−13.4)	14.0 (12.9−15.2)	9.2 (7.9−10.7)	8.9 (7.54−10.4)	14.2 (13.1−15.4)
Elevated HbA1c (pre‐diabetic *plus* diabetic)	39.1 (37.8−40.4)	41.1 (39.4−42.7)	35.1 (32.8−37.4)	34.3 (32.2−36.6)	41.5 (39.8−43.1)
**Comorbidities**					
Hypertensive and/or elevated HbA1c (>5.7%)	53.0 (50.6−53.4)	53.2 (51.5−54.9)	49.5 (47.0−52.0)	46.3 (43.9−48.9)	54.8 (53.1−56.5)
Comorbidity (hypertension and prediabetes)	6.1 (5.45−6.8)	6.3 (5.5−7.2)	5.8 (4.7−7.1)	4.5 (3.5−5.7)	6.9 (6.1−7.8)
Comorbidity (hypertension and diabetes)	3.5 (3.0−4.0)	3.6 (3.0−4.3)	3.2 (2.4−4.2)	1.6 (1.1−2.4)	4.4 (3.7−5.1)

Abbreviations: BMI, body mass index; DBP, diastolic blood pressure; DSD, differentiated service delivery; HbA1c, glycosylated haemoglobin A1c; SBP, systolic blood pressure; SD, standard deviation.

^a^ Systolic blood pressure: ≤120 mmHg and/or diastolic blood pressure ≤80 mmHg.

^b^ Systolic blood pressure: 120−139 mmHg and/or diastolic blood pressure 80/89 mmHg.

^c^ Systolic blood pressure: 140−159 mmHg and/or diastolic blood pressure 90−99 mmHg.

^d^ Systolic blood pressure: ≥ 160 mmHg and/or diastolic blood pressure ≥100 mmHg.

### 3.2 HIV status

The results show that 32.6% of participants had been on ART for 10 years or more. Regarding the HIV regimen, 96.3% of participants were on the dolutegravir‐based regimen. No significant differences were observed between males and females, nor between rural and urban areas.

### 3.3 Physical measurements

Overall, 22.2% of the surveyed participants were overweight, while 10.5% were classified as obese. Among females, 25.8% were overweight, compared to 14.9% of males. Furthermore, 23.6% of PLHIV residing in urban areas and 19.3% in rural areas were overweight. In terms of obesity, 13.7% of the female PLHIV were obese compared to 4.1% of the male PLHIV. Additionally, 8.0% of rural residents and 11.7 % of urban residents were classified as obese.

### 3.4 Blood pressure measurements

The prevalence of hypertension (systolic blood pressure [SBP]≥140 mmHg and/or diastolic blood pressure [DBP]≥90 mmHg) was 22.5%. Males had a higher prevalence than females, at 23.7% versus 21.9%. In urban areas, the prevalence was 24.1% compared to 19.0% in rural areas. Among the hypertensives, 48.4% were unaware of their condition. This includes 44.8% of females, 55.1% of males, 50.2% in rural areas and 47.7% in urban areas. In terms of hypertension treatment, 66.1% of individuals were not on antihypertensive medications; specifically, 61.7% of females, 74.6% of males, 68.1% in rural areas and 65.4% in urban areas. Of those on medication, 30.6% were controlled, with a minor difference between females and males, at 30.9% and 29.7%, respectively. Additionally, 32.5% of the cases were controlled in rural areas, compared to 29.9% in urban areas. The study further found that the rate of hypertension among those in the age range of 30–44 years was 17.1% (Supplementary Table A1) and that this rate increases to as high as 45.0% in PLHIV who are 60 years or older.

### 3.5 Glycosylated HbA1c measurements

The mean HbA1c was 5.6% (SD = 1.2%). The prevalence of PLHIV with an HbA1c above 5.7% was 39.1%, with a higher prevalence among females (41.1%) compared to males (35.1%). The proportion of participants classified as prediabetic was 26.7%. The differences between females and males, as well as rural and urban participants, were 27.0%, 25.8%, 25.5% and 27.2%, respectively. The highest rates of prediabetes were observed in the 45–59 age group (29.5%). Those in the 18–29 and 30–44 age groups had a prevalence of 27.2% and 27.6%, respectively. PLHIV identified as diabetic accounted for 12.5%. More females (14.0%) than males (9.2%) were found to be diabetic. The study also found that (Supplementary Table A2) diabetes mellitus among PLHIV aged 18–29 (11.4%) was similar to that of those aged 30–44 (11.8%), with this rate increasing rapidly to 22.0% among those aged 60 years or older.

### 3.6 Comorbidity of hypertension and elevated HbA1c among PLHIV

The prevalence of the comorbidity involving hypertension and diabetes mellitus (HbA1C >6.5%) was 3.5%. The prevalence was significantly higher in urban areas (4.4%) than in rural areas (1.6%), and was almost the same between males and females (3.6% vs. 3.2%). Furthermore, 6.1% had a comorbidity of hypertension and prediabetes (HbA1c 5.7−6.4%). Female PLHIV had a higher prevalence (6.3%) compared to males (5.8%). The prevalence was higher in urban areas (6.9%) compared to rural areas (4.5%). Overall, the study found that 53.0% of the surveyed PLHIV had either hypertension or prediabetes or diabetes or a combination of hypertension and prediabetes or diabetes mellitus. The prevalence was higher in females (53.2%) than in males (49.5%). More than half resided in urban areas (54.8%) compared to rural areas (46.3%).

### 3.7 Associations between hypertension and common risk factors

Table 3 presents factors independently associated with hypertension among PLHIV. Those aged 30–44 years (PR = 2.1), 45–49 years (PR = 3.3) and 60 years or older (PR = 4.7) compared to those aged 18–29 (*p*<0.001); married or cohabiting individuals (PR = 1.3), and widowed, divorced or separated individuals (PR = 1.4) compared to those never married (*p* = 0.03); being overweight (PR = 1.4) and obese (PR = 1.9) compared to normal weight (*p*<0.001) was associated with hypertension. Time spent on ART, consumption of raw salt or salty sauces, elevated total cholesterol, diabetes and differentiated service delivery (DSD) were not associated with hypertension in the multivariable model.

**Table 3:** Factors independently associated with the prevalence of hypertension

		Univariable model (weighted)		Multivariable model (weighted)	
Factors	# (%) with hypertension (unweighted) *n* = 1083	PR^*^ (95% CI)	*p*‐value sig = 0.05^***^	PR (95% CI)	*p*‐value sig = 0.05***
**Age group (years)**					
18−30	48 (6.9)	Ref	<0.001	Ref	<0.001
30−44	370 (17.15)	2.5 (1.8−3.3)		2.1 (1.5−2.9)	
45−59	553 (30.5)	4.4 (3.3−5.9)		3.3 (2.4−4.7)	
60+	188 (45.0)	6.52 (4.8−8.8)		4.7 (3.3−6.8)	
**Marital status**					
Never married	73 (10.7)	Ref		Ref	0.03
Married/cohabitating	656 (22.8)	2.1 (1.7−2.7)	<0.001	1.3 (1.0−1.7)	
Widowed/divorced/separated	428 (28.1)	2.7 (2.1−3.5)		1.4 (1.1−1.8)	
**Education**					
No formal schooling	22 (9.6)	Ref	<0.001	Ref	0.001
Less than primary	86 (11.0)	0.8 (0.6−1.0)		0.8 (0.6−1.1)	
Primary	162 (14.0)	0.7 (0.6−0.9)		0.8 (0.6−1.0)	
Secondary	257 (12.1)	0.8 (0.6−1.0)		0.9 (0.7−1.1)	
College/university	104 (23.5)	1.1 (0.9−1.4)		1.1 (0.9−1.5)	
**Body mass index (kg/m^2^)**					
Healthy weight	552(19.3)	Ref	<0.001	Ref	<0.001
Underweight	61 (13.6)	0.7 (0.5−0.9)		0.7 (0.5−0.9)	
Overweight	317 (27.9)	1.5 (1.3−1.7)		1.4 (1.2−1.5)	
Obesity	213 (37.4)	2.1(1.8−2.4)		1.9 (1.6−2.1)	
**Time on ART**					
≤1 year	130 (17.3)	Ref	<0.001	^**^	
2−5 years	274 (18.8)	1.0 (0.9−1.3)			
6−9 years	286 (23.6)	1.3 (1.1−1.6)			
10+ years	472 (28.0)	1.6 (1.3−1.9)			
**Differentiated service delivery (DSD)**					
Non‐DSD (≤180 days HIV drug supply)	245 (19.2)	Ref	0.003	^**^	
DSD (180 days HIV drug supply)	908 (24.0)	1.2 (1.1−1.4)			
ARV regimen					
AZT + FTC/3TC + ATV‐r	2 (50.0)	Ref	<0.001	Ref	0.03
FDC (TDF + FTC/3TC + EFV)	10 (18.2)	1.7 (0.5−5.6)		1.6 (0.5−5.5)	
TAF + FTC/3TC + DTG	199 (39.8)	3.3 (1.2−10.3)		2.2 (0.8−6.1)	
TLD (TDF + FTC/3TC + DTG)	924 (21.0)	1.9 (0.6−5.5)		1.8 (0.6−1.1)	
Diabetic					
No	439 (12.2)	Ref	<0.001	^**^	
Yes	182 (17.3)	1.3 (1.1−.15)			
Total cholesterol					
Normal (≤5.18 mmol/l)	1019 (22.4)	Ref	0.001	^**^	
Borderline (5.19−56.19 mmol/l)	49 (36.3)	1.5 (1.2−1.9)			
High (≥6.20 mmol/l)	14 (34.1)	1.5 (1.0−2.3)			
Raw salt or salty sauce consumption					
No	781 (23.6)	Ref	0.04	^**^	
Yes	381 (21.1)	0.9 (0.8−1.0)			

* Prevalence ratio.

** Dropped out *p*>0.1.

*** Wald‐test.

### 3.8 Associations between diabetes mellitus and common risk factors

Factors associated with diabetes mellitus among PLHIV are shown in Table 4. College or university‐educated PLHIV (PR = 2.1) compared to those with no formal education; individuals aged 60 years or older (PR = 1.4; *p*<0.001), in comparison to those aged 18–29 years; and those with high (≥6.2 mmol/l) total cholesterol (PR = 2.2; *p* = 0.005), as opposed to normal total cholesterol (<5.2 mmol/l), as well as being overweight (PR = 1.4) or obese (PR = 1.6) compared to those with normal weight, showed a significant association (*p*<0.001) with diabetes mellitus. In the multivariable model, hypertension was not linked to diabetes mellitus.

**Table 4:** Factors independently associated with the prevalence of diabetes mellitus

		Univariable model (weighted)		Multivariable model (weighted)	
	# (%) with diabetes mellitus (unweighted)	PR (95% CI)	*p*‐value sig = 0.05^***^	PR (95% CI)	*p*‐value sig = 0.05^***^
**Age group**					
18−30	74 (11.4)	Ref	<0.001	Ref	
30−44	237 (11.8)	0.9 (0.7−1.2)		0.9 (0.7−1.3)	0.007
45−59	233 (13.9)	1.1 (0.8−1.4)		0.9 (0.7−1.3)	
60+	84 (22.0)	1.7 (1.3−2.3)		1.4 (1.0−2.0)	
**Marital status**					
Never married	85 (13.0)	Ref	0.001	Ref	0.03
Married/cohabitating	320 (12.1)	0.9 (0.7−1.1)		0.9 (0.7−1.2)	
Widowed/divorced/separated	224 (15.9)	1.2 (0.9−1.5)		1.1 (0.8−1.5)	
**Education**					
No formal schooling	67 (28.5)	Ref	<0.001	Ref	<0.001
Less than primary	195 (22.3)	1.1 (0.7−1.8)		1.1 (0.7−1.8)	
Primary	256 (20.4)	1.4 (0.9−2.2)		1.4 (0.9−2.1)	
Secondary	498 (21.8)	1.2 (0.8−1.9)		1.2 (0.8−1.9)	
College or university	145 (31.5)	2.4 (1.5−3.7)		2.1 (1.3−3.4)	
**Body mass index**					
Healthy weight	297 (11.1)	Ref		Ref	<0.001
Under weight	52 (12.1)	^**^	<0.001	1.2 (0.9−1.6)	
Overweight	168 (16.1)	1.4 (1.2−1.7)		1.4 (1.1−1.6)	
Obesity	106 (20.8)	1.8 (1.4−2.2)		1.6 (1.3−2.0)	
**Time on ART**					
≤1 year	73 (10.8)	Ref	<0.001	Ref	0.02
2−5 years	159 (12.1)	1.1 (0.8−1.4)		1.0 (0.8−1.4)	
6−9 years	128 (11.2)	0.9 (0.7−1.2)		0.9 (0.6−1.1)	
10+ years	271 (17.0)	1.5 (1.1−1.9)		1.2 (0.9−1.6)	
**ARV regimen**					
AZT + FTC/3TC + ATV‐r	2 (50.0)	Ref	<0.001	Ref	0.06
FDC (TDF + FTC/3TC + EFV)	10 (18.2)	1.5 (0.4−5.6)		1.2 (0.3−4.0)	
TAF + FTC/3TC + DTG	199 (39.8)	1.9 (0.6−5.4)		1.3 (0.5−3.7)	
TLD (TDF + FTC/3TC + DTG)	924 (21.0)	1.1 (0.4−3.1)		1.0 (0.4−2.7)	
**Total cholesterol**					
Normal (≤5.18 mmol/l)	1019 (94.2)	Ref	<0.001	Ref	0.005
Borderline (5.19−56.19 mmol/l)	49 (4.5)	1.3 (0.9−1.9)		1.1 (0.7−1.7)	
High (≥6.20 mmol/l)	14 (1.3)	2.4 (1.5−3.9)		2.2 (1.4−3.6)	
**Hypertensive**					
No	439 (12.2)	Ref	<0.001	^**^	
Yes	182 (13.4)	1.3 (1.1−1.50)			
**Physical exercise**					
Work‐related vigorous intensity exercise					
Yes	110 (10.1)	Ref	<0.001	Ref	0.01
No	521 (14.3)	1.5 (1.2−1.8)		1.3 (1.0−1.6)	
Walk or cycle					
Yes	213 (14.4)	Ref	0.04	Ref	0.03
No	418 (12.8)	1.2 (1.0−1.4)		1.2 (1.0−1.4)	

** Dropped out *p*>0.1.

*** Wald‐test.

## 4 DISCUSSION

We estimated the prevalence of hypertension and diabetes mellitus and their associated risk factors among PLHIV on ART in Zambia. To our knowledge, this is the first study nationally to use a representative sample to estimate the prevalence of hypertension and diabetes mellitus, along with their associated risk factors, among PLHIV. Our study's data collection approach was comprehensive, as we employed the complete WHOSTEPs survey questionnaire [40], previously utilized only in the general population, and the adapted ZAMPHIA questionnaire [2] to assess the prevalence of hypertension and diabetes mellitus, along with their risk factors, in a nationally representative sample of PLHIV in Zambia. To ensure accurate blood pressure readings, we adhered to the standardized protocol, which involves three blood pressure measurements with a 5‐minute rest between each, as recommended by WHO guidelines [41]. This method was necessary to minimize the influence of short‐term variability and the white coat effect, thus producing a more reliable estimate of resting blood pressure [45]. Furthermore, in LMIC settings where ambulatory or community‐based blood pressure monitoring remains limited [46], our approach proved to be a more reliable alternative.

Our findings show that more than half (53.0%) of the PLHIV in Zambia have either hypertension, prediabetes, diabetes or both (hypertension and diabetes or prediabetes). Hypertension prevalence among PLHIV in Zambia was found to be very high at 22.5%. This finding is consistent with other studies done in SSA [47, 48, 49, 50, 51, 52, 53]. The findings are higher than the prevalence of 14.7% reported in a similar survey conducted in Zambia that used blood pressure measurements in the HIV electronic medical record system to determine the prevalence [54]. An indication that the national electronic medical record system used to monitor and manage the HIV programme in Zambia may be underestimating the burden of hypertension among PLHIV in care. The prevalence is also higher than the 19.1% found in the general population in Zambia by the WHOSTEPs survey [39], further concretizing evidence that PLHIV have a higher prevalence of hypertension than the general population. The findings are also consistent with the conclusions of a recent meta‐analysis on NCDs in SSA [55]. A study in South Africa found that the prevalence of hypertension among older PLHIV was 50.1% [56]. In our study, the prevalence of hypertension among those who were 45 years or older was 75.5% (see Supplementary Table A1). Furthermore, the presence of hypertension in 17% of the PLHIV in the age range of between 30 and 44 years may suggest the early onset of hypertension among PLHIV in Zambia and would imply the need for a lower age cut‐off for intensified screening for hypertension.

Our results further show that nearly half of the hypertensive PLHIV in Zambia are not aware of their condition. A systematic review and meta‐analysis reported that the awareness of hypertension diagnosis among PLHIV in SSA was at 28.4% [57]. Despite urban areas having access to a higher distribution of health facilities, over‐the‐counter pharmacies, including trained medical staff and equipment available compared to rural areas, only one‐third of hypertensive PLHIV are on medication. There is an urgent need to conceptualize, operationalize and fully support evidence‐based population‐level interventions to reduce high blood pressure [41]. One starting point would be the comprehensive implementation of the (contextually adapted) WHO Package of Essential Noncommunicable Disease Intervention for Primary Health Care (WHOPEN) in rural and urban areas [58], WHO‐HEARTs [59] and the WHO hypertension treatment in adults guidelines [41], coupled with applicable HIV/NCD care services integration models. If no effective intervention measures are implemented, other studies have projected that the prevalence could reach as high as 46% by 2030 in the region [60].

Evidence shows that 5–10% of prediabetic persons eventually develop diabetes, and that without effective interventions such as lifestyle modification, 70% develop diabetes [61]. In Zambia, our study found that prediabetes affects females and males PLHIV equally in both rural and urban areas and that the prevalence is also high among those aged 18–29 years old. Given the disproportionality in the health system's capacity to respond to NCDs, especially in rural areas [62], prediabetic PLHIV are at increased risk of diabetes‐associated morbidity and mortality without even knowing that they have the condition due to the limited availability of spot blood sugar and HbA1c testing capacities at the primary healthcare level.

More than a tenth of our study participants had HbA1c results that were suggestive of diabetes mellitus. The estimated prevalence rate was higher than that estimated by other studies conducted in SSA [63, 64, 65], but lower than in a nationally representative study conducted in South Africa [66]. We also found the prevalence of diabetes to be significantly higher in women and among the overweight and obese PLHIV, similar to the findings of a study in Tanzania that used HbA1c to establish the prevalence of glucose impairment [67]. The study also identified an association between diabetes mellitus and higher education levels at the college or university stage. This phenomenon can be attributed to the contextual specifics of college or university formal jobs in Zambia, which are predominantly white‐collar jobs. Moreover, these occupations are primarily located in urban areas [68], and evidence shows that they are associated with sedentary lifestyles, reduced physical activity and increased consumption of processed foods [68, 69, 70]. Testing rates for spot blood sugar, as well as confirmatory tests via HbA1c, remain low, especially at the primary healthcare level in SSA [71]. A study in Malawi found that, despite being in contact with a health facility every 3–6 months to access HIV care, the prevalence of undiagnosed diabetes mellitus among PLHIV remained high [72]. There is a need to routinize the testing of blood glucose at the primary healthcare level to ensure early detection and treatment of diabetes mellitus, through the application of the standardized guidelines such as the WHO‐HEARTs‐D [73] and the WHOPEN [58].

Obesity plays a critical role in developing cardiovascular and cardiometabolic conditions [74, 75, 76]. Our results show that one in four of the PLHIV who were obese were also diabetic. There is a need to invest in preventive public health initiatives that focus on preventing overweight and obesity to yield improved health outcomes through the reduction of incidence associated with cardiovascular and cardiometabolic complications [77]. Health systems should incorporate physical activity and healthy diets into their public health programmes for HIV and NCDs. Urban planning should incorporate facilities that would encourage increased physical activity among the residents. Integration of HIV and NCD care services should be explored as a possible cost‐effective approach to effectively respond to the rising burden of NCDs in LMICs [78, 79].

### 4.1 Strengths and limitations

To our knowledge, this was the first study in Zambia to determine the national prevalence of hypertension and elevated blood sugar among PLHIV. It had a large sample size and was weighted based on the burden of HIV at the district level. The results are generalizable to the general HIV population in Zambia. Surveyed PLHIV were randomly selected from well‐established ART clinics country wide. The study also had several limitations that are worth mentioning. First, the study did not capture the recent viral load for every participant. The study also did not collect random or fasting blood sugar to determine spot blood sugar levels, instead relying more on historical blood sugar levels as measured by HbA1c. However,HbA1c has been shown to underestimate the prevalence of diabetes in PLHIV on ART [80, 81]. Secondly, evidence suggests a cyclic association between tuberculosis (TB) and hyperglycaemia [82, 83, 84], possibly contributing to the explanation for high HbA1c levels (>5.7%) reported in this study. However, the study did not assess TB status, thereby limiting understanding of its role in the observed elevated HbA1c in the surveyed population.

## 5 CONCLUSIONS

This is the first nationally representative study conducted on a large pool of PLHIV accessing care in HIV clinics in both rural and urban areas across Zambia to determine the prevalence of hypertension and diabetes mellitus and their associated risk factors. Our results highlight a high prevalence of both hypertension and diabetes mellitus in both males and females in rural and urban areas of Zambia. To reverse this rising burden of hypertension and diabetes mellitus among PLHIV, there is an urgent need for investment in evidence‐based interventions that support cost‐effective integrated HIV and NCD care for PLHIV.

## COMPETING INTERESTS

The authors declare no competing interests.

## AUTHOR CONTRIBUTIONS

CM conceptualized the manuscript, helped develop the methodology, analysed the data and led the writing of the original draft. SS, MM, MPC, AM, CZ, MS, RC and PS helped develop the methodology. SB led the development of the methodology and supervised the data analysis. HP, HH and JS provided supervisory support. WM conceptualized the manuscript with CM, helped develop the method, reviewed and edited the final manuscript. All authors read and approved the manuscript.

## FUNDING

This study was funded by the Global Fund to Fight AIDS, Tuberculosis and Malaria Office at the Ministry of Health, Lusaka, Zambia.

## Supporting information




**Table A1**. Background characteristics among hypertensive PLHIV (unweighted).
**Table A2**. HbA1c test category by background characteristic (unweighted).

## Data Availability

We will require all interested parties to provide the data request in writing. Once agreed to, data transfer agreements will be developed. All data transfers must comply with all applicable Zambian regulatory requirements.
