# Supplementary material for: Prevalence and risk factors of hypertension and diabetes among persons living with HIV in Zambia: results of a national facility‐based cross‐sectional survey
Source: J Int AIDS Soc. 2025 Oct 7;28(10):e70051. doi: 10.1002/jia2.70051 (PMC12501770; doi:10.1002/jia2.70051)
Supplement: Supplementary file 1 — Table A1. Background characteristics among hypertensive PLHIV (unweighted). Table A2. HbA1c test category by background characteristic (unweighted). [file JIA2-28-e70051-s001.docx]

**Supplementary tables**

**Table A1. Background characteristics among hypertensive PLHIV (unweighted)**

|  | **Number of participants (% of column total)** | **Normal (% of row total)** | **Hypertensive (% of row total)** | **Chi-square** |
| --- | --- | --- | --- | --- |
| Overall | N= 5108 | 3946 (77.2) | 1162 (22.7) |  |
| Age group |  |  |  |  |
| 18-29 | 699 (13.7) | 651 (93.1) | 48 (6.9) | <0.001 |
| 30-44 | 2158 (42.4) | 1788 (82.8) | 370 (17.15) |  |
| 45-59 | 1813 (35.6) | 1260 (69.5) | 553 (30.5) |  |
| 60+ | 418 (8.2) | 230 (55.0) | 188 (45.0) |  |
| Education level |  |  |  |  |
| No formal schooling | 235 (4.6) | 168 (71.5) | 67 (28.5) | <0.001 |
| Less than primary | 873 (17.1) | 678 (77.7) | 195 (22.3) |  |
| Primary | 1254 (24.6) | 998 (79.6) | 256 (20.4) |  |
| Secondary | 2283 (44.7) | 1785 (78.2) | 498 (21.8) |  |
| College/University | 460 (9.0) | 315 (68.5) | 145 (31.5) |  |
| Marital status |  |  |  |  |
| Never married | 684 (13.4) | 611 (89.3) | 73 (10.7) | <0.001 |
| Currently married or cohabiting | 2882 (56.6) | 2226 (77.2) | 656 (22.8) |  |
| Divorced, separated or widowed | 1523 (29.9) | 1095 (71.9) | 428 (28.1) |  |
| BMI |  |  |  |  |
| Underweight (<18.5 Kg/m^2^) | 450 (9.0) | 389 (86.4) | 61 (13.6) | <0.001 |
| Normal weight (18.6 to 24.9 Kg/m^2^) | 2860 (57.0) | 2308 (80.7) | 552 (19.3) |  |
| Overweight (25 to 29.9 Kg/m^2^) | 1137 (22.7) | 820 (72.1) | 317 (27.9) |  |
| Obesity (>30 Kg/m^2^) | 570 (11.4) | 357 (62.6) | 213 (37.4) |  |
| Current tobacco smoker |  |  |  |  |
| No | 4611 (90.3) | 3565 (77.3) | 1046 (22.7) | 0.74 |
| Yes | 497 (9.7) | 381 (76.7) | 116 (23.3) |  |
| Current alcohol consumer |  |  |  |  |
| No | 275 (18.3) | 212 (77.1) | 63 (22.9) | 0.39 |
| Yes | 1230 (81.7) | 918 (74.6) | 375 (25.4) |  |
| Raw salt or salty sauce consumption |  |  |  |  |
| No | 3306 (64.7) | 2525 (76.4) | 781 (23.6) | 0.04 |
| Yes | 1802 (35.3) | 1421 (78.9) | 381 (21.1) |  |
| Time on ART (years) |  |  |  |  |
| ≤ 1 year | 750 (14.7) | 620 (82.7) | 130 (17.3) | <0.001 |
| 2-5 years | 1457 (28.5) | 1183 (81.2) | 274 (18.8) |  |
| 6-9 years | 1214 (23.8) | 928 (76.4) | 286 (23.6) |  |
| 10+ years | 1687 (33.0) | 1215 (72.0) | 472 (28.0) |  |
| Differential Service Delivery (DSD) |  |  |  |  |
| Non-DSD (≤180 days HIV drug supply) | 1274 (25.1) | 1029 (80.8) | 245 (19.2) | 0.001 |
| DSD (180 days HIV drug supply) | 3795 (74.9) | 2887 (76.0) | 908 (24.0) |  |
| ARV regimen |  |  |  |  |
| Dolutegravir based regimen | 4904 (96.0) | 3781 (77.1) | 1123 (22.9) | 0.207 |
| Other | 204 (4.0) | 165 (80.9) | 39 (19.1) |  |
| Normal (≤5.18 mmol/l) | 1019 (94.2) | 3520 (77.5) | 1019 (22.5) | <0.001 |
| Borderline (5.19-56.19 mmol/l) | 49 (4.5) | 86 (63.7) | 49 (36.3) |  |
| High (≥6.20 mmol/l) | 14 (1.3) | 27 (65.8) | 14 (34.2) |  |
| Diabetic |  |  |  |  |
| No | 4021 (86.6) | 3151 (78.4) | 870 (21.6) | <0.001 |
| Yes | 621 (13.4) | 439 (70.7) | 182 (29.3) |  |
| Work-related physical (vigorous) activity |  |  |  |  |
| No | 3924 (76.8) | 3022 (77.0) | 902 (23.0) | 0.46 |
| Yes | 1184 (23.2) | 924 (78.0) | 260 (22.0) |  |
| Cycle or walk regularly |  |  |  |  |
| No | 1533 (30.0) | 1164 (75.9) | 369 (24.1) | 0.14 |
| Yes | 3575 (70.0) | 2782 (77.8) | 793 (22.2) |  |
| Leisure related vigorous activity |  |  |  |  |
| No | 4478 (87.7) | 3461 (77.3) | 1017 (22.7) | 0.86 |
| Yes | 630 (12.3) | 485 (77.0) | 145 (23.0) |  |

***column percent**

**Table A2. HbA1c test category by background characteristic (unweighted)**

| **Characteristic** | **Number of participants (% of column total)** | **Normal (% of row total)** | **Prediabetes (% of row total)** | **Diabetes (% of row total)** | **Chi-square** |
| --- | --- | --- | --- | --- | --- |
| Overall | N = 4734 | 2804 (59.2) | 1299 (27.4) | 631 (13.3) |  |
| Age group |  |  |  |  |  |
| 18-29 | 651 (13.8) | 400 (61.4) | 177 (27.2) | 74 (11.4) | <0.001 |
| 30-44 | 2011 (42.6) | 1258 (62.6) | 516 (25.7) | 237 (11.8) |  |
| 45-59 | 1677 (35.5) | 950 (56.7) | 494 (29.5) | 233 (13.9) |  |
| 60+ | 381 (8.1) | 192 (50.4) | 105 (27.6) | 84 (22.0) |  |
| Education |  |  |  |  |  |
| No formal schooling | 230 (4.9) | 145 (63.0) | 63 (27.4) | 22 (9.6) | <0.001 |
| Less than primary | 779 (16.5) | 488 (62.6) | 205 (26.3) | 86 (11.0) |  |
| Primary | 1153 (24.4) | 685 (59.4) | 306 (26.5) | 162 (14.0) |  |
| Secondary | 2128 (44.9) | 1272 (59.8) | 599 (28.1) | 257 (12.1) |  |
| College/University | 442 (9.3) | 213 (48.2) | 125 (28.3) | 104 (23.5) |  |
| Marital Status |  |  |  |  |  |
| Never married | 653 (13.8) | 392 (60.0) | 176 (27.0) | 85 (13.0) | 0.007 |
| Married/Cohabiting | 2654 (56.3) | 1612 (60.7) | 722 (27.2) | 320 (12.1) |  |
| Divorced/widowed/separated | 1408 (29.9) | 790 (56.1) | 394 (28.0) | 224 (15.9) |  |
| Body Mass Index (kg/m^2^) |  |  |  |  |  |
| Underweight (<18.5) | 431 (9.3) | 265 (61.5) | 114 (26.4) | 52 (12.1) | <0.001 |
| Normal weight (18.6 to 24.9) | 2666 (57.3) | 1664 (62.4) | 705 (26.4) | 297 (11.1) |  |
| Overweight (25 to 29.9) | 1041 (22.4) | 582 (55.9) | 291 (27.9) | 168 (16.1) |  |
| Obesity (>30) | 510 (11.0) | 242 (47.4) | 162 (31.8) | 106 (20.8) |  |
| Current tobacco smoker |  |  |  |  |  |
| No | 4282 (90.4) | 2506 (58.5) | 1190 (27.8) | 586 (13.7) | 0.007 |
| Yes | 452 (9.6) | 298 (65.9) | 109 (24.1) | 45 (10.0) |  |
| Current alcohol consumer |  |  |  |  |  |
| No | 262 (19.2) | 159 (60.7) | 75 (28.6) | 28 (10.7) | 0.32 |
| Yes | 1103 (80.8) | 680 (61.6) | 310 (28.1) | 113 (10.2) |  |
| Time on ART (Years) |  |  |  |  |  |
| ≤ 1 | 676 (14.3) | 418 (61.8) | 185 (27.4) | 73 (10.8) | <0.001 |
| 2-5 | 1318 (27.8) | 823 (62.4) | 336 (25.5) | 159 (12.1) |  |
| 6-9 | 1146 (24.2) | 705 (61.5) | 313 (27.3) | 128 (11.2) |  |
| 10+ | 1594 (33.7) | 858 (53.8) | 465 (29.2) | 271 (17.0) |  |
| Differential Service Delivery (DSD) |  |  |  |  |  |
| Non-DSD (≤180 days HIV drug supply) | 1183 (25.2) | 779 (65.8) | 269 (22.7) | 135 (11.4) | <0.001 |
| DSD (180 days HIV drug supply) | 3513 (74.8) | 2008 (57.2) | 1012 (28.8) | 493 (14.0) |  |
| ARV regimen |  |  |  |  |  |
| Dolutegravir based regimen | 4559 (96.3) | 2727 (59.8) | 1220 (26.8) | 612 (13.4) | <0.001 |
| Other | 175 (3.7) | 77 (44.0) | 79 (45.1) | 19 (10.9) |  |
| **Total cholesterol** |  |  |  |  |  |
| Normal (≤5.18 mmol/l) | 4860 (96.3) | 2705 (59.4) | 1254 (27.5) | 594 (13.0) | 0.004 |
| Borderline (5.19-56.19 mmol/l) | 143 (2.8) | 77 (56.6) | 36 (26.5) | 23 (16.9) |  |
| High (≥6.20 mmol/l) | 46 (0.9) | 20 (46.5) | 9 (20.9) | 14 (32.6) |  |
| **Hypertensive** |  |  |  |  |  |
| No | 3590 (77.3) | 2168 (60.4) | 983 (27.4) | 439 (12.2) | <0.001 |
| Yes | 1052 (22.7) | 576 (54.7) | 294 (27.9) | 182 (13.4) |  |
| Work-related physical (vigorous) activity |  |  |  |  |  |
| No | 3647 (77.0) | 2118 (58.1) | 1008 (27.6) | 521 (14.3) | 0.001 |
| Yes | 1087 (23.0) | 686 (63.1) | 291 (26.8) | 110 (10.1) |  |
| Cycle or walk regularly |  |  |  |  |  |
| No | 3258 (68.8) | 1927 (59.1) | 913 (28.0) | 418 (12.8) | 0.20 |
| Yes | 1476 (31.2) | 877 (59.4) | 386 (26.1) | 213 (14.4) |  |
| Leisure related vigorous activity |  |  |  |  |  |
| No | 4156 (87.8) | 2465 (59.3) | 1127 (27.1) | 564 (13.6) | 0.24 |
| Yes | 578 (12.2) | 339 (58.6) | 172 (29.8) | 67 (11.6) |  |
